# Supplementary material for: QFR Predicts the Incidence of Long-Term Adverse Events in Patients with Suspected CAD: Feasibility and Reproducibility of the Method
Source: J Clin Med. 2020 Jan 14;9(1):220. doi: 10.3390/jcm9010220 (PMC7020025; doi:10.3390/jcm9010220)
Supplement: Supplementary file 1 [file jcm-09-00220-s001.pdf]

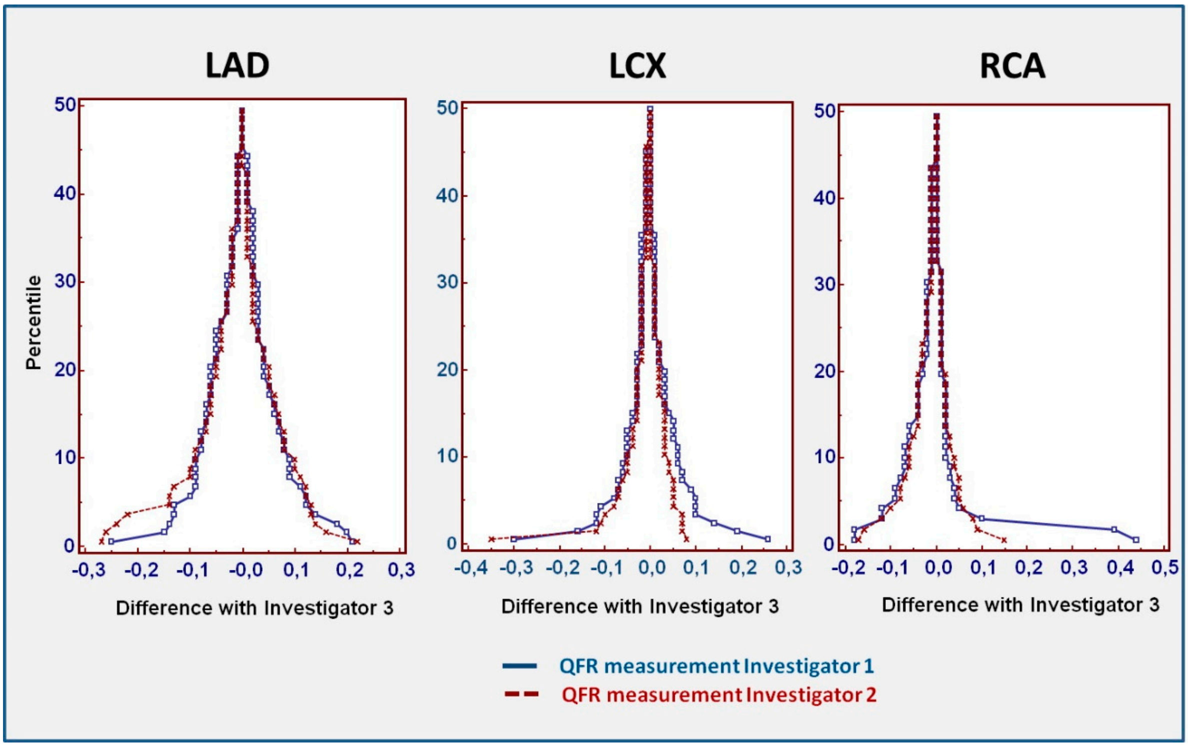

Supplemental Figure S1. Mountain Plots of QFR reproducibility.

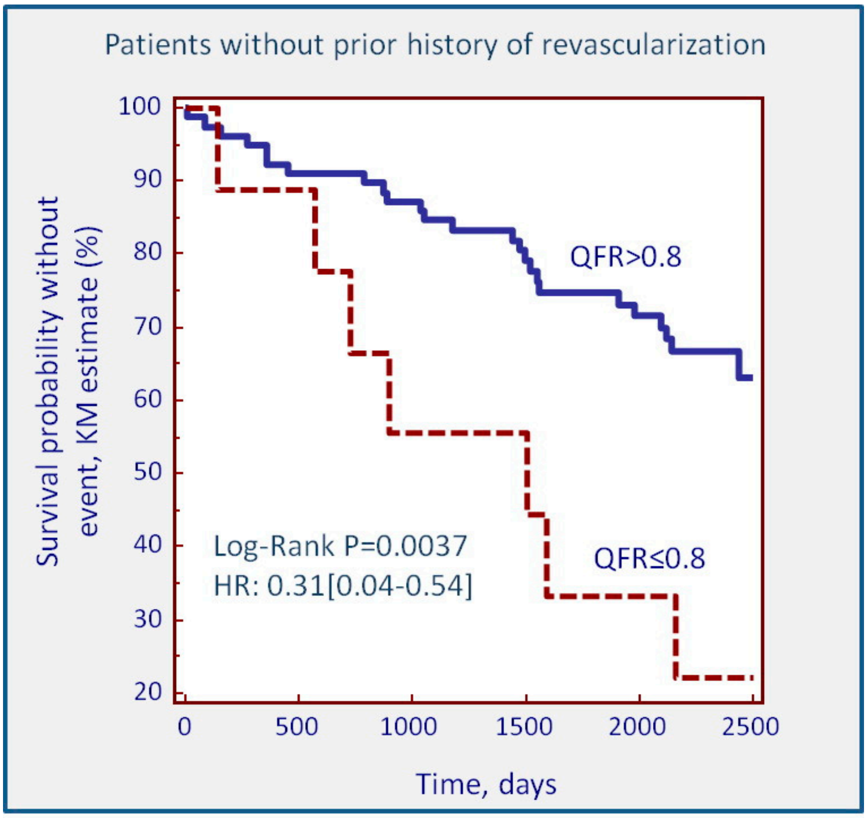

Supplemental Figure S2. QFR Kaplan-Meier event-free survival curves of patients without a prior history of revascularization.

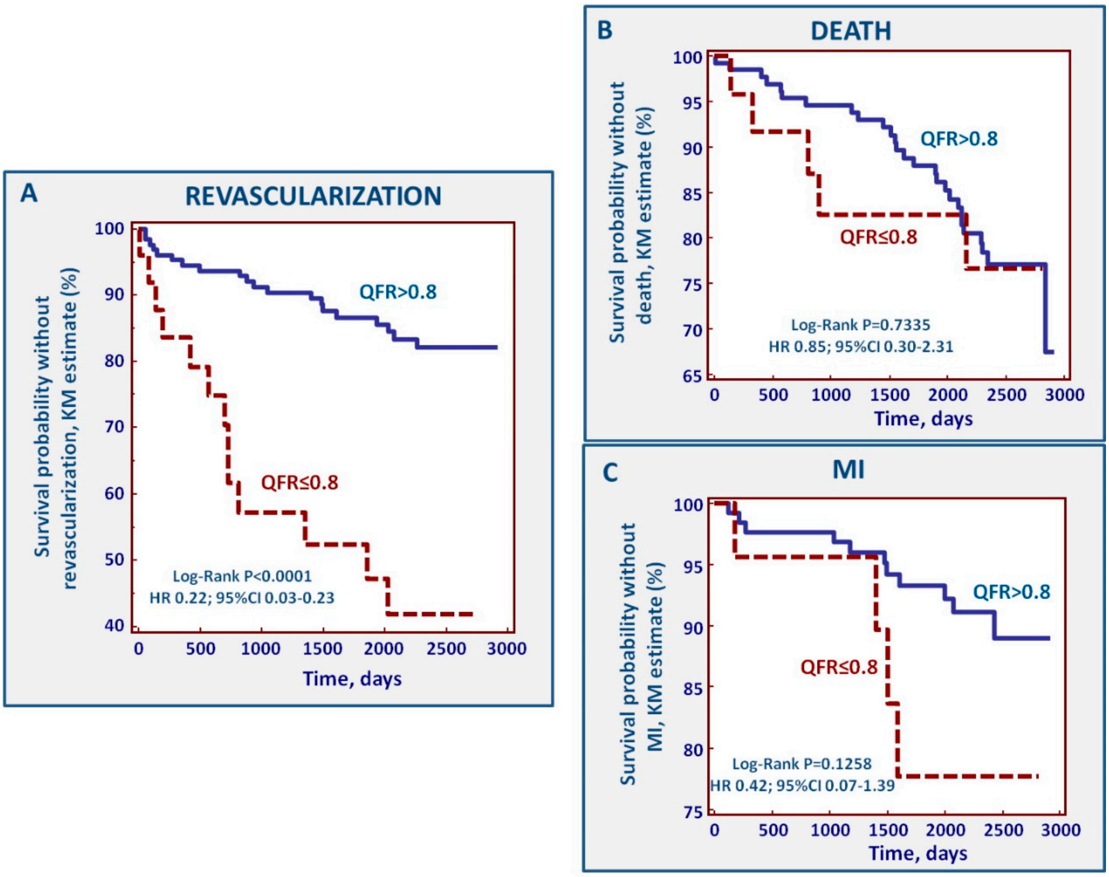

Supplemental Figure S3. Kaplan-Meier curves describing event-free survival of QFR≤0.80 concerning individual endpoints.

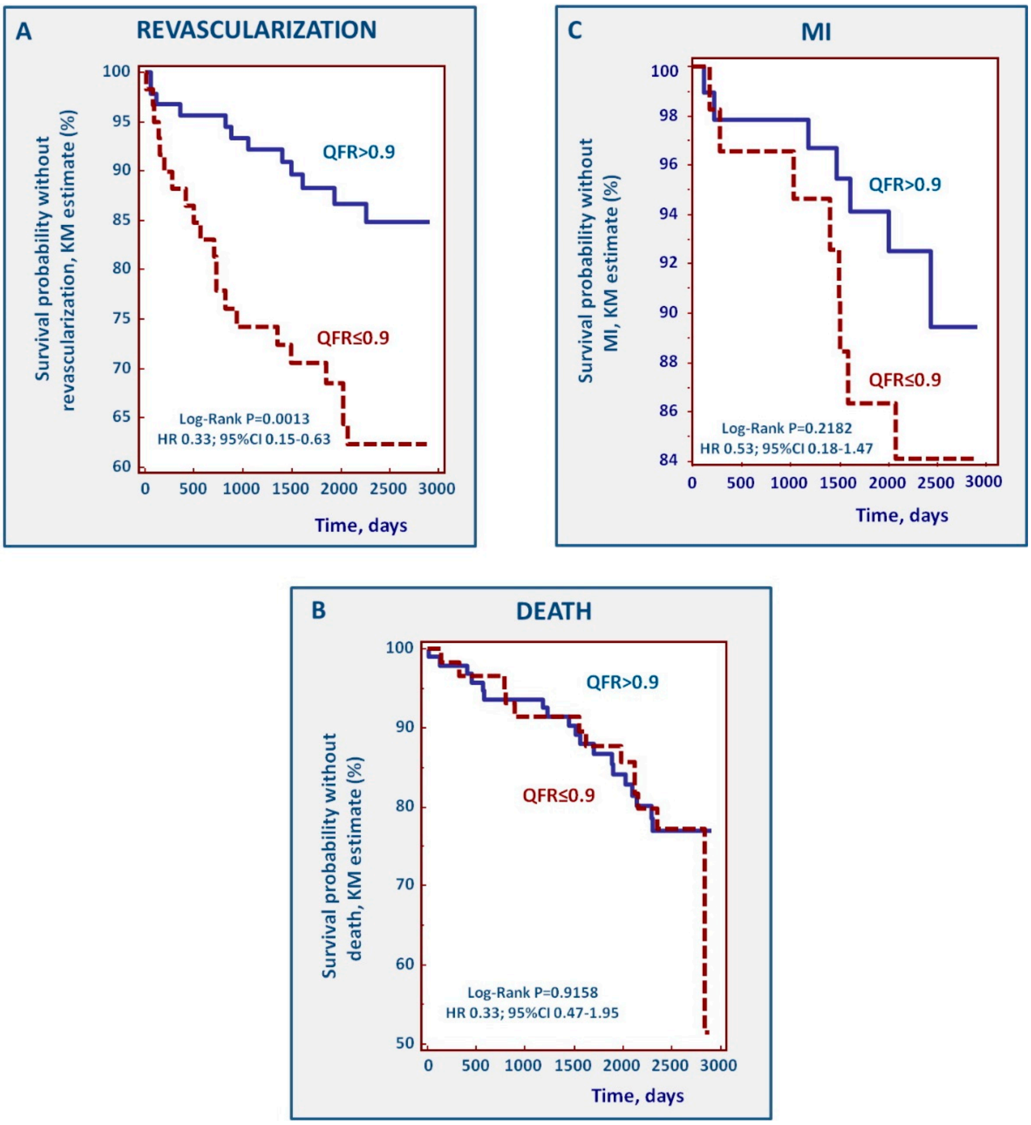

Supplemental Figure S4. Kaplan-Meier curves describing event-free survival of QFR≤0.90 concerning individual endpoints.
